# Supplementary figures and images for: Differential memory enrichment of cytotoxic CD4 T cells in Parkinson’s disease patients reactive to α-synuclein
Source: NPJ Parkinsons Dis. 2025 May 14;11:127. doi: 10.1038/s41531-025-00981-6 (PMC12078614; doi:10.1038/s41531-025-00981-6)

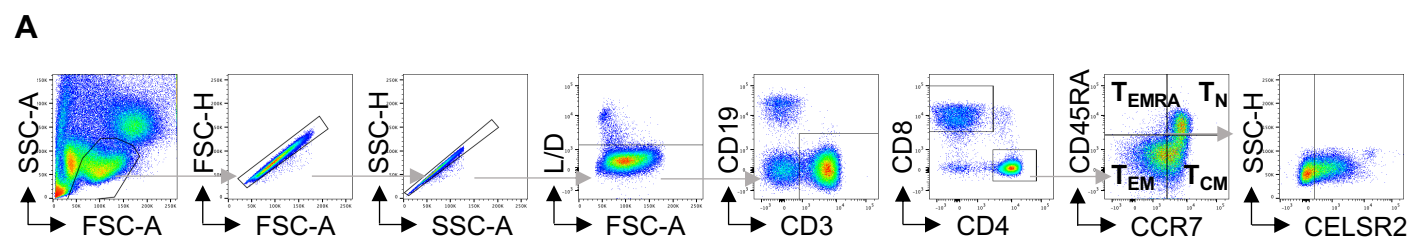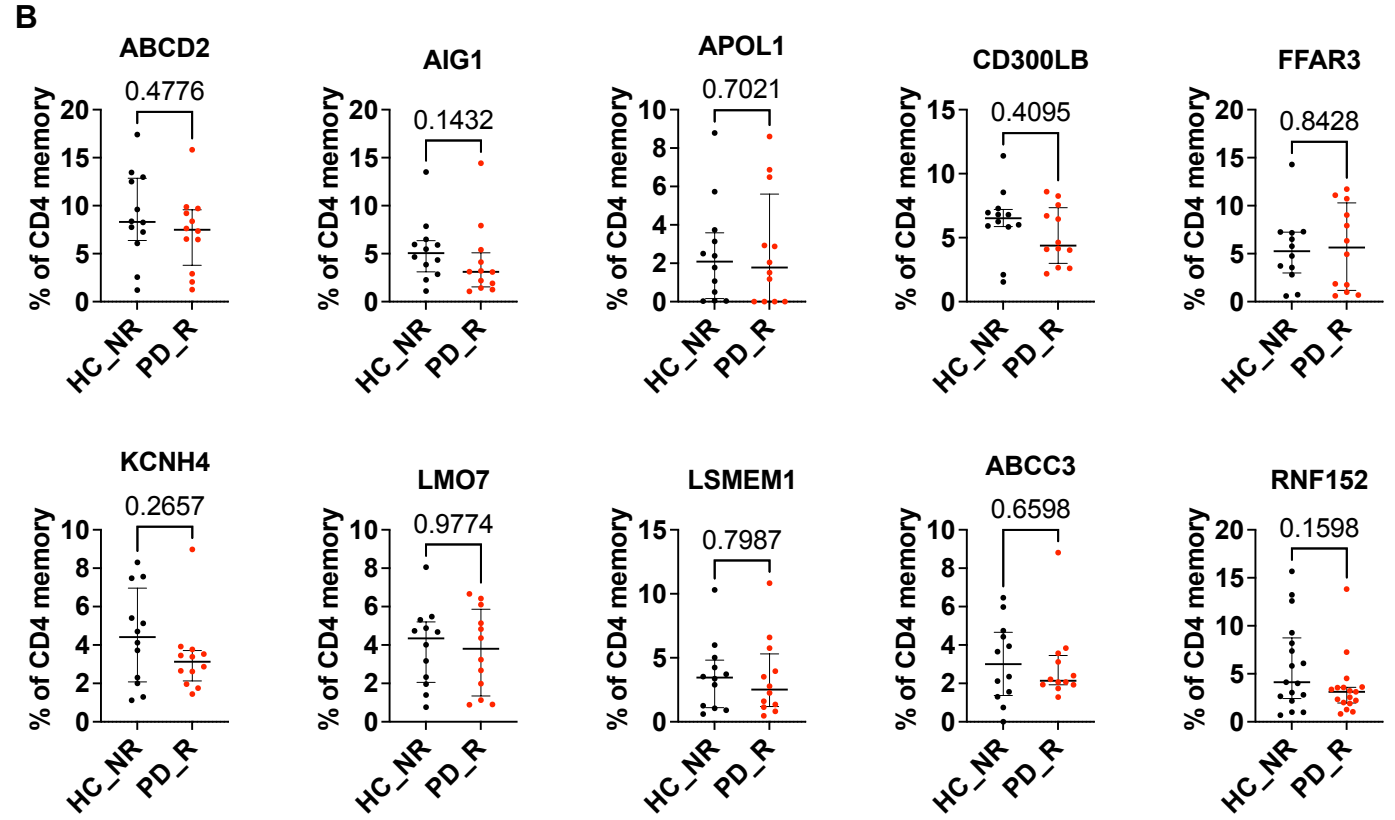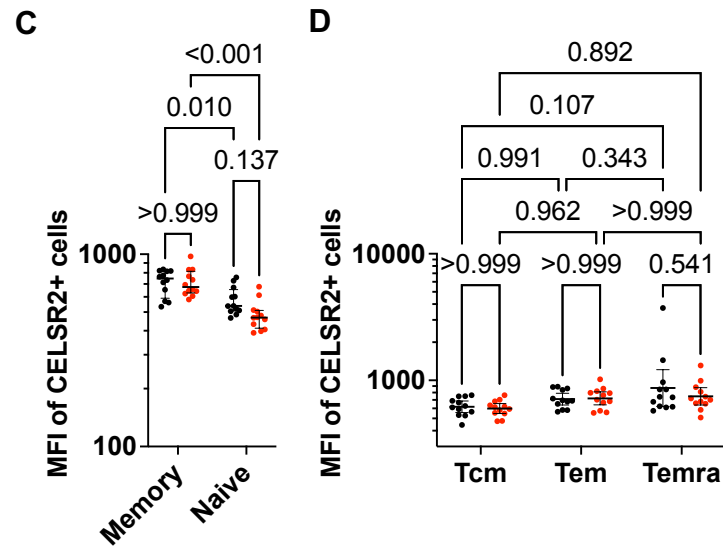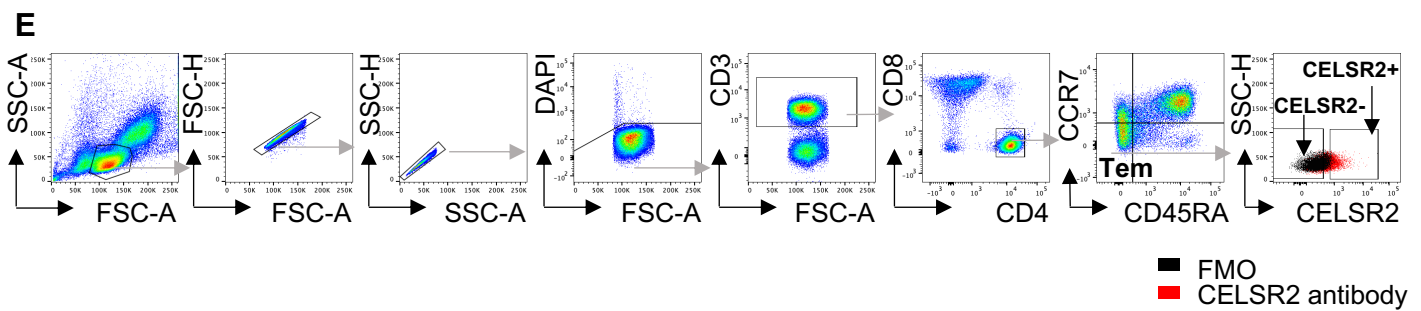

Supplement: Supplementary file 1 — Supplementary Figure 1 [file 41531_2025_981_MOESM1_ESM.pdf]

**A**

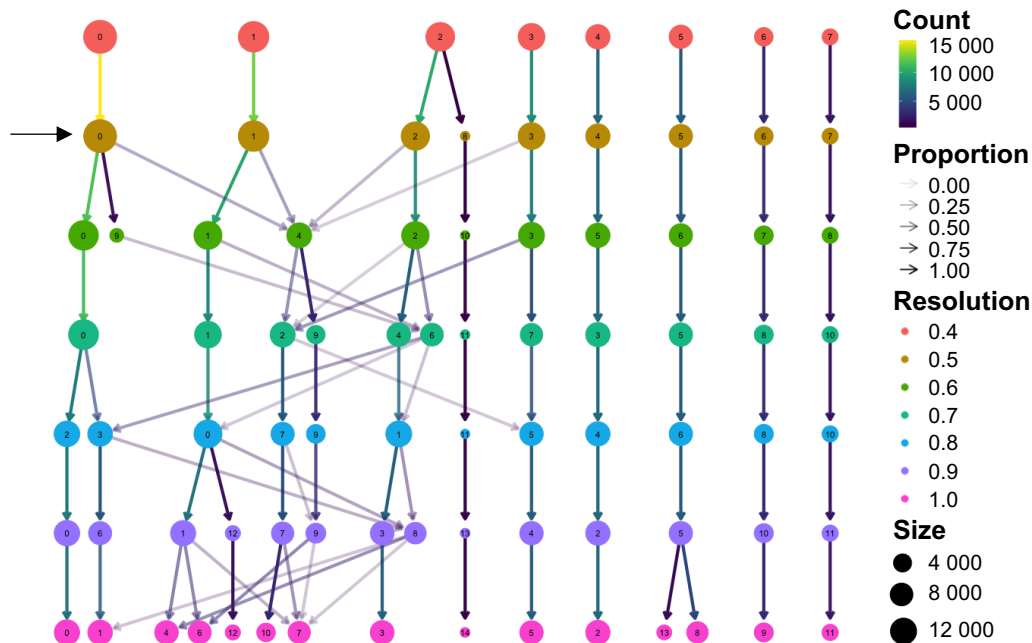

**B**

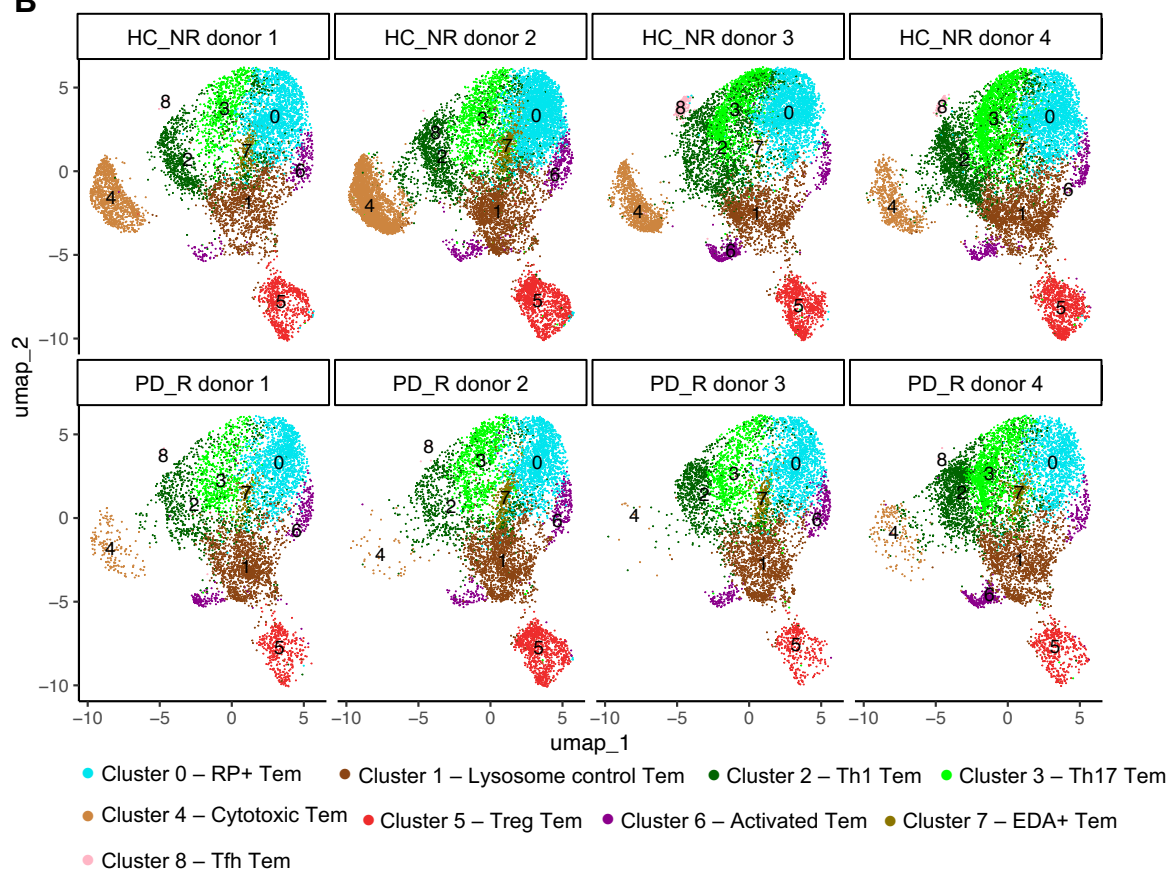

Supplement: Supplementary file 2 — Supplementary Figure 2 [file 41531_2025_981_MOESM2_ESM.pdf]

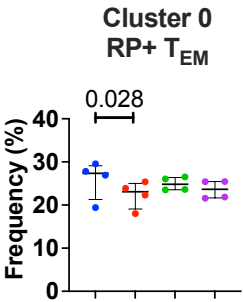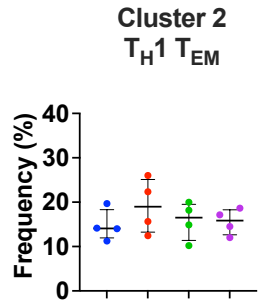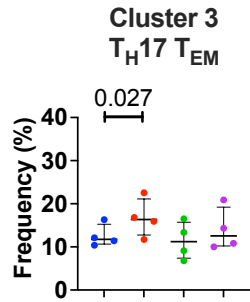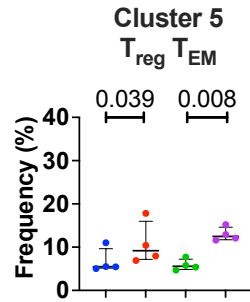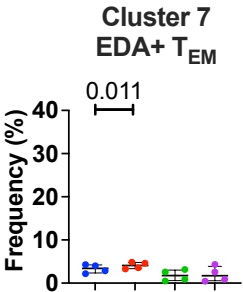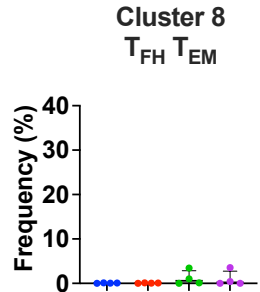

- PD\_R CELSR2+
- PD\_R CELSR2-
- HC\_NR CELSR2+
- HC\_NR CELSR2-

Supplement: Supplementary file 4 — Supplementary Figure 4 [file 41531_2025_981_MOESM4_ESM.pdf]

**A**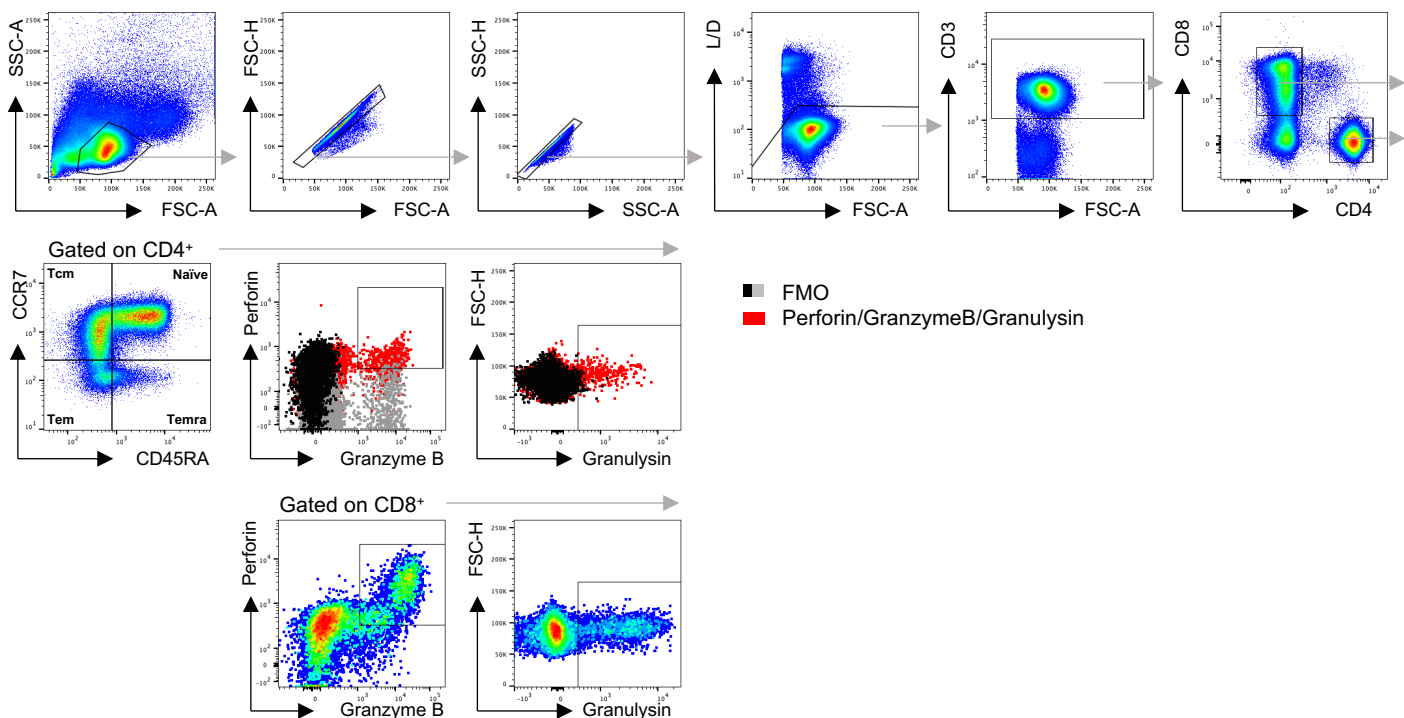**B**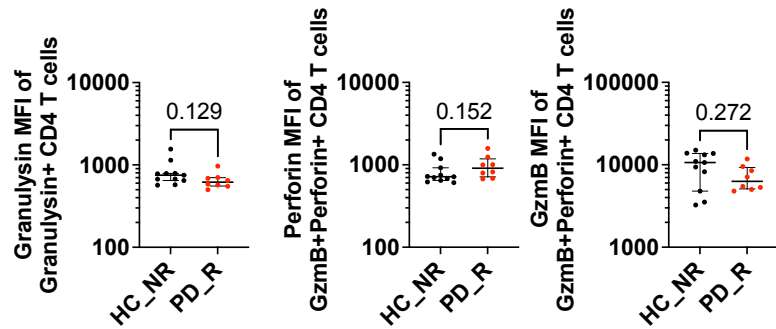**C**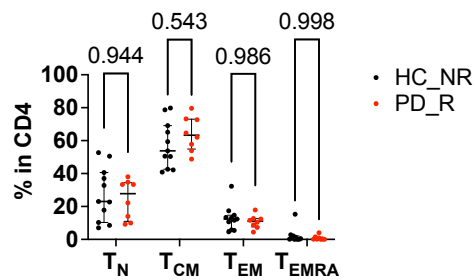**D**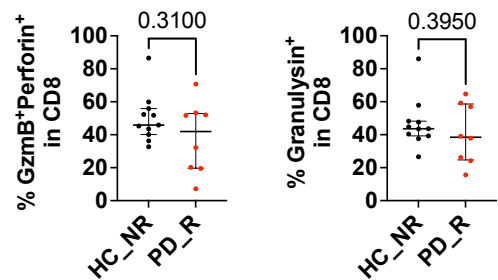**E**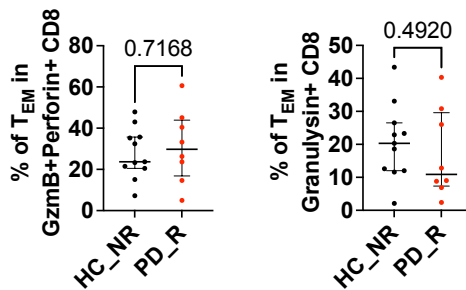

Supplement: Supplementary file 6 — Supplementary Figure 6 [file 41531_2025_981_MOESM6_ESM.pdf]
