# Supplementary material for: Differential memory enrichment of cytotoxic CD4 T cells in Parkinson’s disease patients reactive to α-synuclein
Source: NPJ Parkinsons Dis. 2025 May 14;11:127. doi: 10.1038/s41531-025-00981-6 (PMC12078614; doi:10.1038/s41531-025-00981-6)

Cluster 0 – RP^{+} T_{EM}

Ribosomal gene signature

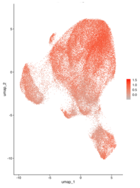

Cluster 1 –Differentiating T_{EM}

NR4A3 *FHIT* *SLC9A9* *SEMA4A*

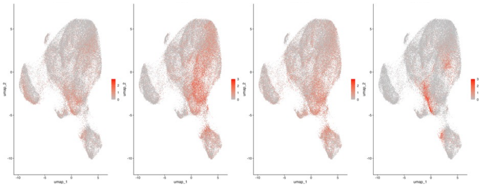

Cluster 2 – T_H1 T_{EM}

GZMK *DUSP2* *KLRB1* *ITGA4*

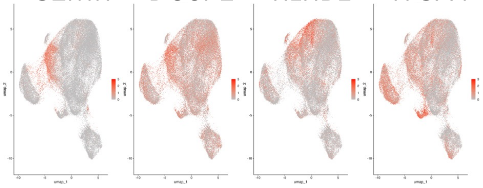

Cluster 3 – T_H17 T_{EM}

IL4I1 *KLRB1* *CCR6*

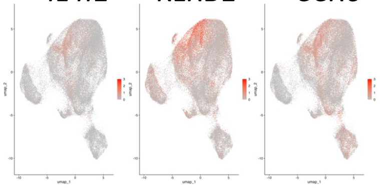

Cluster 4 – Cytotoxic T_{EM}

GZMB *GZMH* *GNLY* *PRF1*

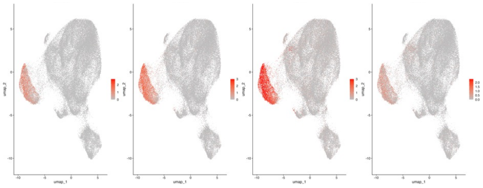

Cluster 5 – T_{reg} T_{EM}

IKZF2 *IL2RA* *CTLA4* *TIGIT*

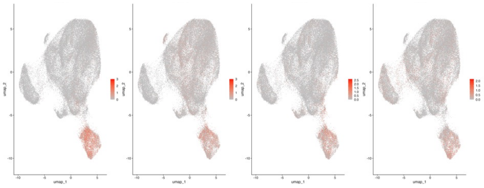

Cluster 6 – Activated T_{EM}

HLA-DRA HLA-DRB1 HLA-DPA1 HLA-DQB1

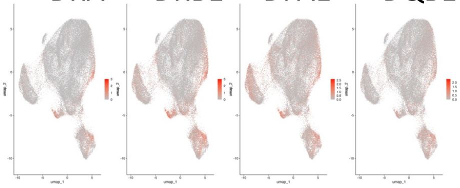

Cluster 5 – EDA^{+} T_{EM}

EDA

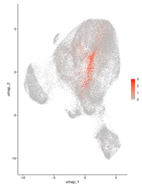

Cluster 7 – T_{FH} T_{EM}

BCL6

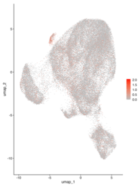

Supplement: Supplementary file 3 — Supplementary Figure 3 [file 41531_2025_981_MOESM3_ESM.pdf]
