# Supplementary material for: Differential memory enrichment of cytotoxic CD4 T cells in Parkinson’s disease patients reactive to α-synuclein
Source: NPJ Parkinsons Dis. 2025 May 14;11:127. doi: 10.1038/s41531-025-00981-6 (PMC12078614; doi:10.1038/s41531-025-00981-6)
Supplement: Supplementary file 5 — Supplementary Figure 5 [file 41531_2025_981_MOESM5_ESM.pdf]

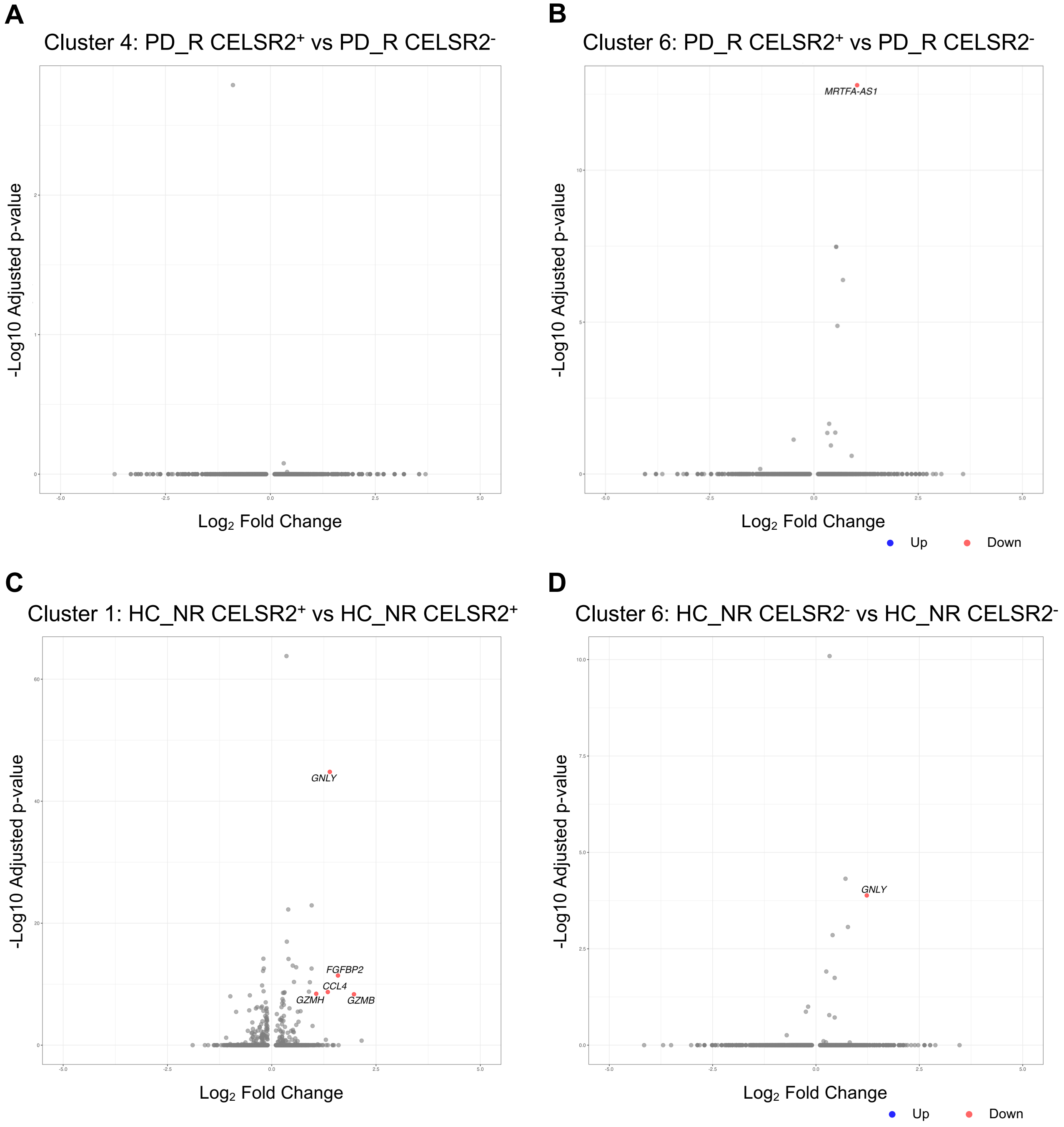
A
Cluster 4: PD_R CELSR2+ vs PD_R CELSR2-
-Log10 Adjusted p-value
Log2 Fold Change
B
Cluster 6: PD_R CELSR2+ vs PD_R CELSR2-
MRTFA-AS1
-Log10 Adjusted p-value
Log2 Fold Change
Up
Down
C
Cluster 1: HC_NR CELSR2+ vs HC_NR CELSR2+
GNLY
FGFBP2
CCL4
GZMH
GZMB
-Log10 Adjusted p-value
Log2 Fold Change
D
Cluster 6: HC_NR CELSR2- vs HC_NR CELSR2-
GNLY
-Log10 Adjusted p-value
Log2 Fold Change
Up
Down
